# Dendronized Polyimides Bearing Long-Chain Alkyl Groups and Their Application for Vertically Aligned Nematic Liquid Crystal Displays

**DOI:** 10.3390/ijms10115031

**Published:** 2009-11-19

**Authors:** Yusuke Tsuda, Jae Min OH, Renpei Kuwahara

**Affiliations:** Department of Biochemistry & Applied Chemistry, Kurume National College of Technology, 1-1-1 Komorino, Kurume, Fukuoka 830-8555, Japan; E-Mails: aligneer@naver.com (J.M.O.); kuwa@mail.cstm.kyushu-u.ac.jp (R.K.); † Ex-senior researcher of SAMSUNG Cheil Industries Inc. in Korea; Present address: Poonglim Apt 418-1801, Jung Ja-Dong, Jang An-Gu, Suwon-Shi, Kyunggi-Do, Korea.; § Present address: Department of Chemistry and Biochemistry, Graduate School of Engineering, Kyushu University, Moto-oka 744, Nishi-ku, Fukuoka 819-0395, Japan.

**Keywords:** polyimide, soluble polyimide, long-chain alkyl group, dendronized polyimide, alignment layers, vertically aligned nematic liquid crystal displays

## Abstract

Polyimides having dendritic side chains were investigated. The terphenylene diamine monomer having a first-generation monodendron, 3,4,5-tris(*n*-dodecyloxy)-benzoate and the monomer having a second-generation monodendron, 3,4,5-tris[-3’,4’,5’-tri(*n*-dodecyloxy)benzyloxy]benzoate were successfully synthesized and the corresponding soluble dendritic polyimides were obtained by polycondensation with conventional tetracarboxylic dianhydride monomers such as benzophenone tertracarboxylic dianhydride (BTDA). The two-step polymerizations in NMP that is a general method for the synthesis of soluble polyimides is difficult; however, the expected dendritic polyimides can be obtained in aromatic polar solvents such as *m-*cresol and pyridine. The solubility of these dendoronized polyimides is characteristic; soluble in common organic solvents such as dichloromethane, chloroform, toluene and THF. These dendronized polyimides exhibited high glass transition temperatures and good thermal stability in both air and under nitrogen. Their application as alignment layers for LCDs was investigated, and it was found that these polyimides having dendritic side chains were applicable for the vertically aligned nematic liquid crystal displays (VAN-LCDs).

## 1. Introduction

Polyimides exhibit excellent thermal and mechanical properties, and have extensive engineering and microelectronics applications [1–5]. Aromatic polyimides such as polyimides based on pyromellitic dianhydride are prepared from aromatic diamines and aromatic tetracarboxylic dianhydrides *via* poly(amic acid)s. Since conventional aromatic polyimides are insoluble, these polymers are usually processed as the corresponding soluble poly(amic acid) precursors, and then either thermally or chemically imidized. However, owing to the instability of poly(amic acid)s and the liberation of water in the imidization process, problems can arise. Extensive research has been carried out to improve the solubility of polyimides and successful recent examples involve the incorporation of fluorine moieties [6–13], bulky side groups [14–15], alicyclic structure [16], spiro linkage groups [17], cardo groups [18–19], pyridine moiety [20–21], porphyrin moiety [22], acridine moiety [23], and sulfonated structure [24–27]. And their applications include as functional materials such as low-k materials [6,7,16], gas transport membranes [8,9,14], materials for organic light-emitting diodes (OLEDs) [18,23], and membranes for polymer electrolyte fuel cells (PEFCs) [24–27]. Soluble polyimides bearing long-chain alkyl groups have also been reported [28–34], and their applications mainly involve their use as alignment layers for liquid crystal displays (LCDs).

Our research group has systematically investigated the synthesis and characterization of soluble polyimides based on alicyclic tetracarboxylic dianhydrides such as 2,3,5-tricarboxycyclopentyl acetic dianhydride (TCA-AH) [35,36], 5-(2,5-dioxotetrahydrofuryl)-3-methyl-3-cyclohexene-1,2-dicarboxylic anhydride (cyclohexene-DA) [37], and 4-(2,5-dioxotetrahydrofuran-3-yl)-tetralin-1,2-dicarboxylic anhydride (tetralin-DA) [37], and aromatic diamines bearing long-chain alkyl groups such as alkyldiaminobenzophenone (ADBP-X, X = carbon numbers of alkyl chain) [38], alkoxydiaminobenzene (AODB-X) [39], diaminobenzoic acid alkylester (DBAE-X) [40], and alkyldiaminobenzamide (ADBA-X) [41]. Our recent paper has described soluble polyimides having dendritic moieties on their side chain [42], and the synthesis of soluble polyimides in ionic liquids was also investigated [43].

In this paper, the synthesis and characterization of a novel series of soluble polyimides having dendritic moieties (dendronized polyimides) based on the copolymerization of aromatic diamines having the first- or second- generation monodendrons bearing long-chain alkyl groups. The attachment of alkyl side chains to polyimides has recently been used to increase pretilt angles generated by the polyimides in alignment layer applications of liquid crystal displays (LCDs) and, thus, these studies are of great practical importance [29–34]. The application for the polyimide alignment layers based on dendronized polyimides for the vertically aligned nematic liquid crystal displays (VAN-LCDs) is finally described.

## 2. Experimental

### 2.1. Materials

The reagents for the synthesis of aromatic diamines were purchased from Tokyo Chemical Industry Co., Ltd. (TCI) or Wako Pure Chemical Industries, Ltd (Wako) and used as received. 3,3′,4,4′-Benzophenone tetracarboxylic dianhydride (BTDA) and 4,4′-diaminodiphenylether (DDE) were purchased from TCI and purified as follows: BTDA, mp. 228.3 °C, recrystallized from acetic anhydride; DDE, mp. 193.6 °C, recrystallized from ethanol. 2,2-Bis(3,4-anhydrodicarboxyphenyl) hexafluoropropane (6FDA) were purchased from DAIKIN Industries, Ltd and used as received. *N*-Methyl-2-pyrrolidone (NMP) (Mitsubishi Chemicals) was distilled under reduced pressure using 4Å molecular sieves. Other conventional reagents were purchased from TCI or Wako and used as received.

### 2.2. Measurements

The inherent viscosities of all polymers were measured using a Cannon Fenske viscometer at a concentration of 0.5 g/dL in NMP at 30 °C. Size exclusion chromatography (SEC) measurements were performed in chloroform at 40 °C with a TOSOH HLC-8020 equipped with a TSK-GEL ALPHA-M column. Number average molecular weight (*Mn*), weight average molecular weight (*Mw*) and polydispersity (*Mw/Mn*) were determined by TOSOH Multi Station GPC-8020 analysis system calibrated with a series of polystyrene standards having narrow polydispersities. The polyimide film samples for the measurement of ATR and differential scanning calorimeter (DSC) were prepared by the following casting method. About five wt % polyimide solution in appropriate solvents such as NMP, chloroform, *m*-cresol on aluminum cup or glass substrate and the solution were slowly evaporated by heating on a hotplate at appropriate temperature (*ca.* 50 °C for chloroform, *ca.* 150 °C for NMP and *m*-cresol) until the films were dried, then the films were dried in a vacuum oven at 100 °C for 12 h. In case the molecular weights of polyimides were lower, the polyimide films were tend to be brittle.

Differential scanning calorimetery (DSC) traces were obtained on a Shimadzu DSC-60 under nitrogen (flow rate 30 mL/min) at a heating rate of 20 °C/min using 30 mg of the film samples and the glass transition temperatures (Tg) were read at the midpoint of the heat capacity jump from the second heating scan after cooling from 250 °C. Thermogravimetric analysis (TGA) was performed on a Shimadzu TGA-50 in air or under nitrogen (50 mL/min) at a heating rate of 10 °C/min using 5 mg of a dry powder sample, and 10% weight loss temperatures (Td_10_) were calculated from the second heating scan after cooling from 250 °C. ^1^H- and ^13^C-NMR spectra were measured on a JEOL JNM-AL400 FT NMR instrument in CDCl_3_ or dimethylsulfoxide-d_6_ with tetramethylsilane (TMS) as an internal standard. IR spectra were recorded on a JASCO FT/IR-470 plus spectrophotometer. ATR Pro 450-S attaching Ge prism was used for the ATR measurements of polyimide films.

LCDs test cell properties were measured at Cheil Ind. Inc., Korea as follows: the polyimide solutions were spin-coated onto ITO glass substrates to a thickness of 0.1 μm, and cured at 210 °C for 10 minutes to produce liquid crystal alignment films. After the liquid crystal alignment films were subjected to a rubbing process, the alignment properties and the pretilt angles of the liquid crystal were measured. The surface of the alignment films were rubbed by means of a rubbing machine, two substrates were arranged anti-parallel to each other in such a manner that the rubbing direction of the each substrates were reverse, and the two substrates were sealed while maintaining cell gaps of 50 μm to fabricate liquid crystal cells. The liquid crystal cells were filled with the liquid crystalline compounds (Merk licristal). The alignment properties of the liquid crystal were observed under an orthogonally polarlized optical microscope. The pretilt angles of the liquid crystal were measured by a crystal rotation method.

In order to examine the electrical properties, the test cells were prepared by the same manner as above except the cell gap, 5 μm. The voltage holding ratios were measured with VHRM 105 (Autronic Melchers). To evaluate the VHR, the applied frequency and voltage was 60 Hz, 1V with pulse of 64 μsec. The voltage versus transmittance and optical response properties, such like contrast ratio, response time, image sticking, etc., were measured using computer-controlled system in conjunction with an tungsten-halogen lamp, a function/arbitrary waveform generator, photomultiplier. The residual DCs were measured by C-V method using impedance analyzer.

### 2.3. Synthesis of dendritic diamine monomers bearing long-chain alkyl groups

#### 2.3.1. Dendritic building blocks

These compounds are based on Percec-type monodendron [44–45]. 3,4,5-tris(*n*-Dodecyloxy)-benzoic acid (12G1-AG) and 3,4,5-tris[3’,4’,5’-tris(*n*-dodecyloxy)]benzoic acid (12G2-AG), and their corresponding acid chlorides (12G1-AG-Cl, 12G2-AG-Cl) were synthesized by the method in the previous paper [44–45]. 4-[3,5-Bis(3-aminophenyl)phenyl]carbonylamino]phenyl 3,4,5-tris(*n*-dodecyloxy)benzyloxy benzoate (12G1-AG-Terphenyldiamine) and 4-[3,5-Bis(3-aminophenyl)phe-nyl]carbonylamino]phenyl 3,4,5-tris[3’,4’,5’-tris(*n*-dodecyloxy)benzyloxy]benzoate (12G2-AG-Terphenyl diamine) were synthesized by the following method *via* condensation reactions with 3,5-dibromobenzoic acid and 4-aminophenol, followed by Suzuki coupling reaction using 3-aminophenyl boronic acid.

#### 2.3.2. 12G1-AG-Dibromo precursor

**Figure 1.**
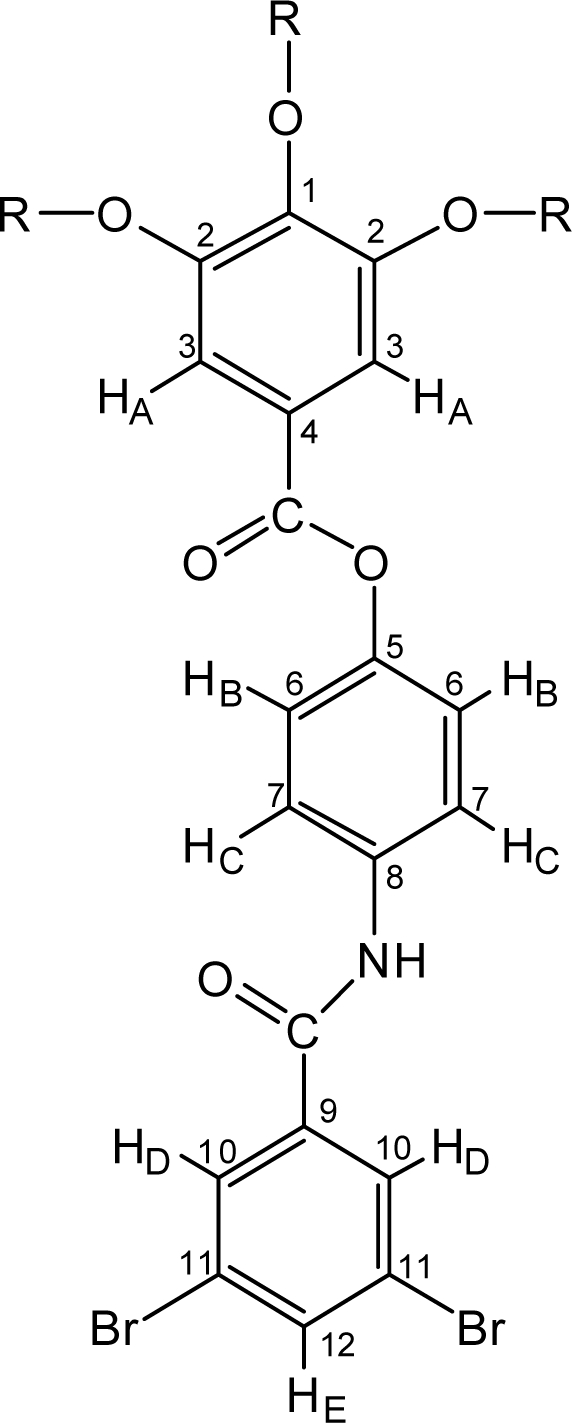
^1^H-NMR assignment of dibromoprecursor (R = −(CH_2_)_11_CH_3_).

2.0 g (7.1 mmol) of 3,5-dibromobenzoic acid and 0.77 g (7.1 mmol) of *p*-aminophenol and a catalytic amount of *N,N*-dimethyl-4-aminopyridine (DMAP, *ca.* 50 mg) were added in 30 mL of THF. A solution of *N,N′*-dicyclohexylcarbodiimide (DCC, 1.47g, 7.1 mmol) in 20 mL of dichloromethane was added dropwise to an ice-cooled above mixture, and the reaction was continued for additional 30 min. Precipitated *N,N′*-dicyclohexylurea (DCU) was filtered off and the residual reaction mixture was added 1.5 mL of triethylamine (TEA), and then added dropwise 4.9 g (7.1 mmol) of 12G1-AG-Cl in 20 mL dichloromethane at room temperature for 30 min. Precipitated TEA-HCl salts were filtered off and the solvent was removed *in vacuo* and the residue was recrystallized from acetone. Yield, 85%. TLC (dichloromethane): *R_f_* = 0.7. Mp 116.2 °C (DSC). ^1^H-NMR (CDCl_3_, δ, ppm) (Figure 1): 0.88 (t, 9H, C*H*_3_, *J* = 6.8 Hz), 1.26 (m, 48H, CH_3_(C*H*_2_)_8_), 1.49 (m, 6H, C*H*_2_CH_2_CH_2_OAr), 1.83 (m, 6H, C*H*_2_CH_2_OAr), 4.06 (m, 6H, C*H*_2_OAr), 7.16 (d, 2H, H_C_, *J* = 8.8 Hz), 7.40 (s, 2H, H_A_), 7.64 (d, 2H, H_B_, *J* = 8.8 Hz), 7.81 (t, 1H, H_E_, *J* = 1.9 Hz), 7.95 (d, 2H, H_D_, *J* = 1.9 Hz), 8.22 (s, 1H, N*H*); ^13^C-NMR (CDCl_3_, δ, ppm): 14.1 (*C*H_3_), 22.7 (CH_3_*C*H_2_), 26.0 (*C*H_2_CH_2_CH_2_OAr), 29.2 (CH_3_(CH_2_)_2_*C*H_2_), 29.6 (CH_3_(CH_2_)_3_(*C*H_2_)_5_), 30.3 (*C*H_2_CH_2_OAr), 31.9 (CH_3_CH_2_*C*H_2_), 69.0 (*C*H_2_OAr, C_2_ positions), 73.4 (*C*H_2_OAr, C_1_ position), 108.2 (C_3_), 121.4 (C_6_), 121.7 (C_7_), 122.9 (C_11_), 123.0 (C_4_), 128.9 (C_10_), 135.0 (C_8_), 136.5 (C_12_), 137.6 (C_9_), 142.7 (C_1_), 147.0 (C_5_), 152.5 (C_2_), 162.6 (Ar*C*ONHAr), 165.5 (Ar*C*O_2_Ar); IR (KBr): 1730, 1640 (C=O), 1190, 1120 (-O-) cm^−1^. *Anal*. Calcd. for C_56_H_85_Br_2_NO_6_: C, 65.42%; H, 8.33%; N, 1.36%. Found: C, 65.33%; H, 8.25%; N, 1.33%.

#### 2.3.3. 12G1-AG-Terphenyldiamine

**Figure 2.**
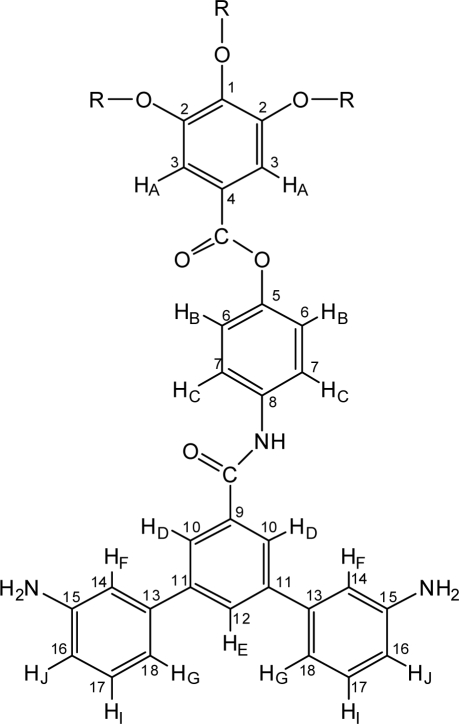
^1^H-NMR assignment of terphenyldiamine (R = − (CH_2_)_11_CH_3_).

1,2-Dimethoxyethane (DME) was bubbled by argon for 10 min before a reaction. To a 100 mL three neck flask, 12G1-AG-Dibromo precursor (2.0 g, 1.9 mmol), DME (30 mL) and 2M Na_2_CO_3_ (5 mL) were added, the reaction mixture was bubbled by argon for 10 min, then Pd(PPh_3_)_4_ (0.2 g, 0.17 mmol) was added. The reaction was continued at reflux temperature (about 80 °C) for 24 h connecting argon balloon. Reaction was monitered by TLC, and the product was appeared (1/1; ethyl acetate/hexane, *R_f_* = 0.3). Water layer was removed by separately funnel and the organic layer was removed by rotary evaporator. To this dark brown residue was added dichloromethane and dried by sodium sulfate. This solution was filtered and evaporated, and the residual mixture was subjected to a column chromatograph (1/1; ethyl acetate/hexane). Fractions were collected by 30 mL each, and after the first fraction came out, the eluent was changed to the mixture solvent (3/2; ethyl acetate/hexane). The sixth fraction was collected and recrystallized from the mixture solvents (1/1; acetone/MeOH). Yield, 60%. TLC (3/2; ethyl acetate/hexane): *R_f_* = 0.4. Mp 164.7 °C (DSC). ^1^H-NMR (CDCl_3_, δ, ppm) (Figure 2): 0.88 (t, 9H, C*H*_3_, *J*=6.8 Hz), 1.26 (m, 48H, CH_3_(C*H*_2_)_8_), 1.47 (m, 6H, C*H*_2_CH_2_CH_2_OAr), 1.80 (m, 6H, C*H*_2_CH_2_OAr), 3.76 (s, 4H, N*H*_2_), 4.05 (t, 6H, C*H*_2_OAr, *J* = 8.8 Hz), 6.69 (d, 2H, H_J_, *J* = 7.6 Hz), 6.87 (s, 2H, H_F_,), 6.99 (d, 2H, H_C_, *J* = 8.2 Hz), 7.20 (m, 4H, H_G_ and H_I_), 7.39 (s, 2H, H_A_,), 7.75 (d, 2H, H_B_, *J* = 8.2 Hz), 7.82 (s, 1H, H_E_,), 7.91 (s, 2H, H_D_,), 8.30 (s, 1H, N*H*); ^13^C-NMR (CDCl_3_, δ, ppm): 14.1 (*C*H_3_), 22.7 (CH_3_*C*H_2_), 26.0 (*C*H_2_CH_2_CH_2_OAr), 29.2 (CH_3_(CH_2_)_2_*C*H_2_), 29.6 (CH_3_(CH_2_)_3_(*C*H_2_)_5_), 30.2 (*C*H_2_CH_2_OAr), 31.9 (CH_3_CH_2_*C*H_2_), 69.0 (*C*H_2_OAr, C_2_ positions), 73.4 (*C*H_2_OAr, C_1_ position), 108.1 (C_3_), 113.4 (C_14_), 114.2 (C_16_), 116.9 (C_18_), 120.9 (C_6_), 121.9 (C_7_), 123.4 (C_4_), 124.1 (C_10_), 128.4 (C_12_), 129.3 (C_17_), 135.1 (C_8_), 135.8 (C_9_), 140.6 (C_11_), 141.4 (C_13_), 142.5 (C_1_), 146.6 (C_2_), 146.7 (C_5_), 152.5 (C_15_), 165.0 (Ar*C*ONHAr), 166.2 (Ar*C*O_2_Ar); IR (KBr): 3370, 3280 (NH_2_), 1730, 1650 (C=O), 1190, 1120 (-O-) cm^−1^. *Anal*. Calcd. for C_68_H_97_N_3_O_6_: C, 77.60%; H, 9.29%; N, 3.99%. Found: C, 77.51%; H, 9.22%; N, 3.93%.

#### 2.3.4. 12G2-AG-Dibromo precursor

0.26 g (0.94 mmol) of 3,5-dibromobenzoic acid and 0.10 g (0.94 mmol) of *p*-aminophenol and catalytic amount of DMAP (*ca.* 10 mg) were added in 20 mL of THF. A solution of DCC (0.20 g, 0.97 mmol) in 5 mL of dichloromethane was added dropwise to an ice-cooled above mixture, and the reaction was continued for additional 30 min. The intermediate product was monitored by TLC (2/1; ethyl acetate/hexane, *R_f_* = 0.6). Precipitated DCU was filtered off and the residual reaction mixture was added 1.5 mL of TEA and added dropwise 2.0 g (0.94 mmol) of 12G2-AG-Cl in 10 mL of dichloromethane at room temperature for 12 h. Precipitated TEA-HCl salts were filtered off and the solvent was removed *in vacuo* and the residue was subjected to column chromatograph as dichloromethane as an eluent. Yield, 65%. TLC (dichloromethane): *R_f_* = 0.7. Mp 116.8 °C (DSC). ^1^H-NMR (CDCl_3_, δ, ppm, TMS): 0.87 (t, 27H, C*H*_3_, *J* = 6.4 Hz), 1.26 (m, 144H, CH_3_(C*H*_2_)_8_), 1.42 (m, 18H, C*H*_2_CH_2_CH_2_OAr), 1.73 (m, 6H, C*H*_2_CH_2_OAr), 3.77 (t, 4H, C*H*_2_OAr, 4-(3′,5′) position, *J* = 6.4 Hz), 3.88 (t, C*H*_2_OAr, 8H, 3,5-(3′,5′) positions, *J* = 6.4 Hz), 3.94 (t, 6H, CH_2_OAr, 3,4,5-(4′) positions, *J* = 6.4 Hz), 5.07 (s, 4H, ArC*H*_2_OAr, 3,5 positions), 5.09 (s, 2H, ArC*H*_2_OAr, 4 position), 6.63 (s, 2H, *ortho* to CH_2_, 4 position), 6.65 (s, 4H, *ortho* to CH_2_, 3,5 positions), 7.18 (d, 2H, H_C_, *J* = 8.8 Hz), 7.53 (s, 2H, H_A_,), 7.67 (d, 2H, H_B_, *J* = 8.8 Hz), 7.83 (t, 1H, H_E_, *J* = 1.7 Hz), 7.91 (s, 1H, N*H*), 7.94 (d, 1H, H_D_, *J* = 1.7 Hz) ), ^13^C-NMR (CDCl_3_, δ, ppm, TMS): 14.1 (*C*H_3_), 22.7 (CH_3_*C*H_2_), 26.2 (*C*H_2_CH_2_CH_2_OAr), 29.4 (CH_3_(CH_2_)_2_*C*H_2_), 29.7 (CH_3_(CH_2_)_3_(*C*H_2_)_5_), 30.4 (*C*H_2_CH_2_OAr), 31.9 (CH_3_CH_2_*C*H_2_), 68.7 (*C*H_2_OAr, 3,4,5-(3’,5’) positions), 68.9 (*C*H_2_OAr, 3,4,5-(4’) positions), 71.5 (ArCH_2_OAr, 3,5 positions), 73.2 (ArCH_2_OAr, 4 position), 105.4 (*ortho* to CH_2_OAr, 3,5 positions), 105.9 (*ortho* to CH_2_OAr, 4 position), 109.8 (C_3_), 121.2 (C_6_), 121.9 (C_7_), 123.1 (C_4_), 128.8 (C_11_), 131.2 (C_10_), 131.9 (C_8_), 135.0 (*ipso* to CH_2_OAr), 136.8 (*para* to CH_2_OAr), 137.4 (C_12_), 137.7 (C_9_), 142.8 (C_1_), 147.1 (C_5_), 152.3 (*meta* to CH_2_OAr, 3,5 positions), 152.7 (*meta* to CH_2_OAr, 4 positions), 152.9 (C_2_), 162.3 (Ar*C*ONHAr), 164.7 (Ar*C*O_2_Ar); IR (KBr): 1710, 1640 (C=O), 1190, 1120 (-O-) cm^−1^. *Anal*. Calcd. for C_149_H_247_Br_2_NO_15_: C, 72.97%; H, 10.15%; N, 0.57%. Found: C, 73.07%; H, 10.38%; N, 0.52%.

#### 2.3.5. 12G2-AG-Terphenyldiamine

DME was bubled by argon for 10 min before a reaction. To a 50 mL three neck flask, 12G2-AG-Dibromoprecursor (1.0 g, 0.41 mmol), DME (20 mL) and 2M Na_2_CO_3_ (5 mL) were added, the reaction mixture was bubled by argon for 10 min, then Pd(PPh_3_)_4_ (0.1 g, 0.09 mmol) was added. The reaction was continued at reflux temperature (about 80 °C), 24 h connecting argon balloon. Reaction was monitored by TLC (1/20; ethylacetate/dichloromethane), the product was appeared at the spot; *R_f_* = 0.4. Water layer was removed by separately funnel and the organic layer was removed by rotary evaporator. To this dark brown solid was added dichloromethane and dried by sodium sulfate. This solution was filtered and evaporated, and the residual mixture was subjected twice to column chromatography (1/20; ethylacetate/dichloromethane, *R_f_* = 0.4). Yield, 30%. Mp 105.2 °C (DSC). ^1^H-NMR (CDCl_3_, δ, ppm): 0.88 (t, 27H, C*H*_3_, *J* = 6.4 Hz), 1.26 (m, 144H, CH_3_(C*H*_2_)_8_), 1.42 (m, 18H, C*H*_2_CH_2_CH_2_OAr), 1.71 (m, 18H, C*H*_2_CH_2_OAr), 3.77 (t, 4H, C*H*_2_OAr, 4-(3′,5′) position, *J* = 6.4 Hz), 3.87–3.95 (m, C*H*_2_OAr, 3,5-(3′,5′) positions, C*H*_2_OAr, 3,4,5-(4′) positions, *NH_2_*), 5.07 (s, 4H, ArC*H*_2_OAr, 3,5 position), 5.09 (s, 2H, ArC*H*_2_OAr, 4 position), 6.63 (s, 2H, *ortho* to CH_2_, 4 position), 6.65 (s, 4H, *ortho* to CH_2_, 3,5 position), 6.73 (d, 2H, H_J_, *J* = 7.6 Hz), 6.97 (s, 2H, H_F_,), 7.06 (d, 2H, H_C_, *J* = 8.2 Hz), 7.21 (m, 4H, H_G_ and H_I_), 7.54 (s, 2H, H_A_,), 7.75 (d, 2H, H_B_, *J* = 8.2 Hz), 7.91 (s, 1H, H_E_,), 7.99 (s, 2H, H_D_,), 8.10 (s, 1H, N*H*); ^13^C-NMR (CDCl_3_, δ, ppm): 14.1 (*C*H_3_), 22.7 (CH_3_*C*H_2_), 26.1 (*C*H_2_CH_2_CH_2_OAr), 29.3 (CH_3_(CH_2_)_2_*C*H_2_), 29.6 (CH_3_(CH_2_)_3_(*C*H_2_)_5_), 30.3 (*C*H_2_CH_2_OAr), 31.9 (CH_3_CH_2_*C*H_2_), 68.6 (*C*H_2_OAr, 4 position), 68.8 (*C*H_2_OAr, 3,5 positions), 73.1 (*C*H_2_OAr, 4 position), 73.8 (*C*H_2_OAr, 3,5 positions), 105.3 (*ortho* to CH_2_OAr, 3,5 positions), 105.8 (*ortho* to CH_2_OAr, 4 position), 109.6 (C_3_), 113.7 (C_14_), 114.5 (C_16_), 117.4 (C_18_), 120.8 (C_6_), 121.9 (C_7_), 124.0 (C_4_), 124.2 (C_10_), 129.6 (C_12_), 131.2 (C_17_), 131.9 (C_8_), 135.5 (*ipso* to CH_2_OAr), 137.3 (*para* to CH_2_OAr), 137.3 (C_9_), 140.8 (C_13_), 142.1 (C_11_), 146.8 (C_1_), 150.6 (C_5_), 152.2 (*meta* to CH_2_OAr), 152.6 (C_15_), 152.8 (C_2_), 164.5 (Ar*C*ONHAr), 165.0 (Ar*C*O_2_Ar); IR (KBr): 3370, 3280 (NH_2_), 1710, 1640 (C=O), 1190, 1120 (-O-) cm^−1^. *Anal*. Calcd. for C_161_H_259_N_3_O_15_: C, 78.07%; H, 10.54%; N, 1.70%. Found: C, 77.74%; H, 10.50%; N, 1.62%.

### 2.4. Preparation of poly(amic acid)s and polyimides

As a typical example of two-step polymerization in NMP, to a 30–100 mL flask was added the mixture of an equimolar amount of tetracarboxylic dianhydride and aromatic diamine. The system was purged with argon and NMP was added. Monomer concentration was kept at 20 wt%. The mixture was stirred at room temperature under argon for 12 h to allow viscosity to increase. The obtained poly(amic acid) solution was diluted with NMP, and polymer concentration was kept at 10 wt%. About one-third portion of the mixture was poured into a large amount of water or methanol. Precipitated poly(amic acid) was filtered, washed with excess methanol and dried at room temperature for 2 days. The residual two-third portion of the poly(amic acid) solution was used for the following polyimide preparation. To this poly(amic acid) solution were added five molar ratio of pyridine and four molar ratio of acetic anhydride to one molar ratio of tetracarboxylic dianhydride. The system was purged with argon and was stirred at 120 °C under argon for 4 h. Powdered polyimide was obtained by precipitation from a large amount of methanol, filtered, and washed with a large amount of methanol, and dried in a vacuum oven at 100 °C for 1 day. Polymerization conditions using solvents such as *m-*cresol and pyridine are mentioned in the next section. The structures of polyimides soluble in chloroform were confirmed by ^1^H-NMR using CDCl_3_ as a solvent. IR spectra were obtained by ATR measurement using the polyimide films samples. The detailed discussions of these characterizations are described in the next section. The obtained ^1^H NMR and IR spectra data are as follows.

*Polyimide Based on BTDA /12G1-AG-Terphenyldiamine /DDE (100/50/50).* IR (ATR): 2925, 2853 (C-H), 1718 (C=O), 1195, 1117 (-O-) cm^−1^.
*Polyimide Based on BTDA /12G1-AG-Terphenyldiamine /DDE (100/25/75)*. IR (ATR): 2924, 2854 (C-H), 1719 (C=O), 1203, 1116 (-O-) cm^−1^.
*Polyimide Based on BTDA /12G2-AG-Terphenyldiamine.* ^1^H-=NMR (CDCl_3_, δ, ppm): 0.76–0.93 (m, C*H*_3_), 1.08–1.51 (m, CH_3_(C*H*_2_)_9_), 1.51–1.87 (m, C*H*_2_CH_2_OAr), 3.57–4.105 (m, C*H*_2_OAr), 6.40–6.80 (m, Ar*H*), 7.07–8.48 (m, Ar*H*); IR (ATR): 2922, 2853 (C-H), 1719 (C=O), 1190, 1110 (-O-) cm^−1^.
*Polyimide Based on BTDA /12G2-AG-Terphenyldiamine /DDE (100/75/25).* ^1^H-NMR (CDCl_3_, δ, ppm): 0.74–0.93 (m, C*H*_3_), 1.11–1.57 (m, CH_3_(CH_2_)_9_), 1.57–1.88 (m, CH_2_CH_2_OAr), 3.51–4.08 (m, C*H*_2_OAr), 6.40–6.77 (m, Ar*H*), 7.08–8.48 (m, Ar*H*); IR (ATR): 2923, 2853 (C-H), 1719 (C=O), 1232, 1115 (-O-) cm^−1^.
*Polyimide Based on BTDA /12G2-AG-Terphenyldiamine /DDE (100/50/50).* IR (ATR): 2924, 2853 (C-H), 1721 (C=O), 1193, 1115 (-O-) cm^−1^.
*Polyimide Based on 6FDA/ 12G1-AG-Terphenyldiamine.* ^1^H-NMR (CDCl_3_, δ, ppm): 0.77–0.93 (m, C*H*_3_), 1.13–1.43 (m, CH_3_(C*H*_2_)_8_), 1.39–1.54 (m, C*H*_2_CH_2_CH_2_OAr), 1.57–1.88 (m, C*H*_2_CH_2_OAr), 3.88–4.14 (m, C*H*_2_OAr), 7.00–7.17 (m, Ar*H*), 7.20–8.05 (m, Ar*H*); IR (ATR): 2925, 2854 (C-H), 1725 (C=O), 1190, 1110 (-O-) cm^−1^.
*Polyimide Based on 6FDA/ 12G2-AG-Terphenyldiamine.* ^1^H-NMR (CDCl_3_, δ, ppm): 0.79–0.98 (m, C*H*_3_), 1.16–1.35 (m, CH_3_(C*H*_2_)_8_), 1.35–1.54 (m, C*H*_2_CH_2_CH_2_OAr), 1.63–1.96 (m, C*H*_2_CH_2_OAr), 3.67–4.11 (m, C*H*_2_OAr), 6.54–6.57 (m, Ar*H*), 7.05–8.17 (m, Ar*H*); IR (ATR): 2922, 2853 (C-H), 1720 (C=O), 1191, 1110 (-O-) cm^−1^.

## 3. Results and Discussion

### 3.1. Monomer synthesis

**Figure 3.**
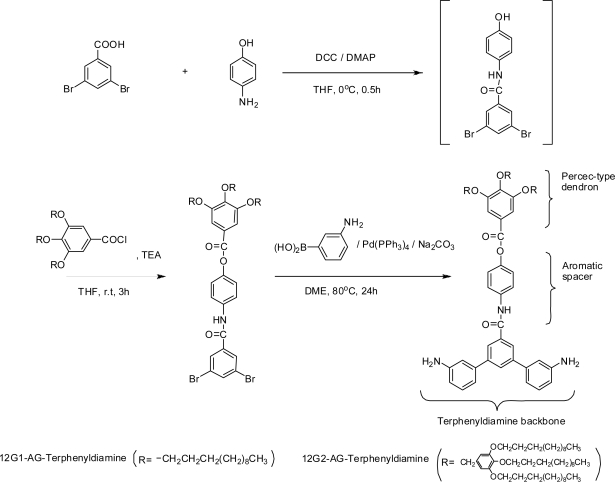
Synthesis of dendritic terphenyl diamine monomers.

4-[3,5-Bis(3-aminophenyl)phenyl]carbonylamino]phenyl 3,4,5-tris(*n*-dodecyloxy)benzyloxy benzoate (12G1-AG-Terphenyldiamine) and 4-[3,5-Bis(3-aminophenyl)phenyl]carbonyl-amino]phenyl 3,4,5-tris[3’,4’,5’-tris(*n*-dodecyloxy)benzyloxy] benzoate (12G2-AG-Terphenyl diamine) were synthesized by the method shown in Figure 3 using the first- and second-generation Percec-type monodendrons.

Firstly, 3,5-dibromo benzoic acid was reacted with 4-aminophenol using DCC/DMAP catalytic system generally used for peptide synthesis. The only product of this condensation reaction was 3,5-dibromo-*N*-(4-hydroxyphenyl)benzamide, because the formation of amide linkage is predominated than the formation of ester linkage under these conditions. Secondly, the acid chlorides (12G1-AG-Cl, 12G2-AG-Cl) were reacted with 3,5-dibromo-*N*-(4-hydroxyphenyl)benzamide using the base catalyst (TEA), and the dibromo-precursors for diamine monomers were easily synthesized. These two reactions can be carried out by a one-pot reaction. Finally, diamine monomers having dendritic moiety were synthesized by Suzuki coupling reaction using 3-aminophenyl boronic acid and a typical Suzuki coupling catalyst, Pd(PPh_3_)_4_.

The structure and the synthetic methods of these diamine monomers having dendritic moiety have several characteristic futures as follows. As dendritic moieties have three long-chain alkyl groups, the enhancement of solubility of polyimides can be expected by the entropy effects of long-chain alkyl groups, and the generation of pretilt angles of liquid crystalline molecules can be expected in case the polyimides using these dendritic diamine monomers are applied for the alignment layers for LCDs. As dendritic moieties are bulky, aromatic spacers are connected for defending steric hindrance. Generally, aliphatic spacers are used for this purpose, however, aliphatic segments tend to lower the thermal stability that is the main feature of polyimides. Therefore, aromatic spacers are selected. A general method for the synthesis of diamine monomers is the reduction of corresponding dinitro precursor using a catalyst such as Palladium on Carbon (Pd/C). However, the second generation of Percec-type monodendron (12G2-AG) contains benzyl ether linkage that is easily cleaved by Pd/C. Consequently, the direct connection of aminophenyl groups using Suzuki coupling reaction are applied, and the terphenyl diamine monomers having the aromatic spacers and Percec-type dendritic moieties are successfully synthesized.

### 3.2. Polymer synthesis

**Figure 4.**
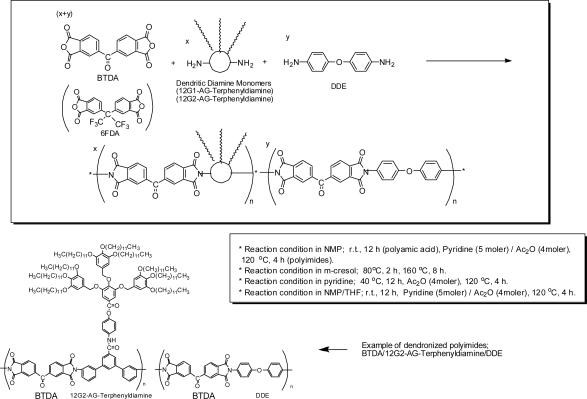
Synthesis of dendronized polyimides based on 12G1-AG-Terphenyldiamine and 12G2-AG-Terphenyldiamines.

The synthesis route for the polyimides and copolyimides based on BTDA, 6FDA, DDE and dendritic diamines bearing long-chain alkyl groups (12G1-AG-Terphenyldiamin, 12G2-AG-Terphenyldiamin) is illustrated in Figure 4, and the results of polymerization are summarized in Table 1.

In our laboratory, two-step polymerization systems consisting of poly(amic acid)s synthesis and chemical imidization have been generally performed. The poly(amic acid)s are obtained by reacting the mixture of an equimolar amount of tetracarboxylic dianhydrides and diamines at room temperature for 12 h under an argon atmosphere. Polyimides were obtained by chemical imidization at 120 °C in the presence of pyridine as base catalyst and acetic anhydride as dehydrating agent. These are the optimized synthesis conditions previously developed for the synthesis of soluble polyimides in our laboratory [35–42]. BTDA that is highly reactive and a common aromatic tetracarboxylic dianhydride is generally used as a dianhydride monomer, and DDE that is highly reactive and a common aromatic diamine is generally used as a diamine co-monomer in case the special functional diamine monomers such as the diamine monomers bearing long-chain alkyl groups are used. In the case of soluble polyimides, clear polyimide solutions are eventually obtained. In other cases, clear poly(amic acid) solutions are obtained, however, gelation or precipitation occur in the course of imidization process.

Thus, the polymerizations of 12G1-AG-terphenyldiamine in NMP were firstly investigated using BTDA, and co-diamine monomer, DDE under above conditions (Table 1). Although viscous poly(amic acid)s solution from the above monomers were obtained, precipitation occurred during the imidization process. It was speculated that the hydrocarbon and phenyl moiety of dendritic diamine monomers reduces the solubility of polyimides in NMP; therefore, a polar aromatic solvent, *m-*cresol, was used to improve the solubility of dendron moieties using a high temperature one-step polymerization system [43,46–48]. The reaction conditions of one-step polymerization system are described in Table 1 and Figure 4. Thus, soluble copolyimides based on BTDA/12G1-terphenyldiamine/DDE (100/50/50) and BTDA/12G1-terphenyldiamine/DDE (100/25/75) were obtained. These copolyimide have high molecular weight (η_inh_ = 0.56–0.78 dL/g) and good film forming ability. On the other hand, homopolyimide based on BTDA/12G1-AG-Terpnenyldiamine was insoluble in *m*-cresol. Solubility of copolyimides, BTDA/12G1-terphenyldiamine/DDE (100/50/50) and BTDA/12G1-terphenyldiamine/DDE (100/25/75), may be improved by the randomizing effect based on copolymerization as well as the entropy effect of long-chain linear alkyl groups. This randomizing effect by copolymerization has been frequently observed in our research [38–41].

According to the previous paper [49] which described the synthesis and properties of polyimides containing multiple alkyl side chains using the diamine monomers such as 2,2-bis{4-[3,4,5-tris(*n*-dodecane-1-yloxy)benzoate]}-4,4’-biphenyldiamine, *m-*cresol cleaved the ester linkages of the alkyl side chains from backbones and *o*-dichlorobenzene or 1-chloronaphthalene was used as one-step polymerization solvents. However, the authors confirmed the existence of long-chain alkyl ether groups in the polyimide based on 12G1-AG-Terphenyldiamine prepared in *m*-cresol by FT-IR (ATR) and TGA measurements described in the next section in detail. In 12G1-AG-Terphenyldiamine monomer, 3,4,5-tris(*n*-dodecyloxy)benzoate group is connected to the polymer backbone through the aromatic spacer (Figure 3), and the ester linkage is separated from the polymer backbone and probably stabilized by the aromatic spacer by the electronic effect. On the other hand, 3,4,5-tris(*n*-alkloxy)benzoate group in aromatic diamine monomers used in ref. 49 are directly connected to the polymer backbone and the connected positions seem to be sterically hindered. Thus, the eater linkages in the side-chains of dendronized polyimides based on BTDA/12G1-AG-Terphenyldiamine are probably more stable against nucleophilic attack by *m*-cresol both sterically and electronically.

The dendronized copolyimides based on the second-generation dendritic monomer (BTDA/12G2-AG-Terphenyldiamine/DDE) were not soluble in even *m-*cresol, and pyridine, an aromatic and more polar solvent, was necessary to dissolve these, probably due to the large aromatic moieties of second-generation dendrons. Thus the soluble hopopolyimide and the copolyimides based on BTDA/12G2-AG-Terphenyldiamine/DDE (100/100/0, 100/75/25, 100/50/50) were obtained in pyridine as a polymerization solvent. Molecular weights of these soluble polyimides were low judged form the value of inherent viscosity (η_inh_ = 0.06–0.22 dL/g). It is spreculated that the huge second-generation dendritic moieties of 12G2-AG-Terphenyldiamine reduce the reactivity of the diamine monomer due to steric hindrance, thus lower the molecular weights of polyimides, even though the aromatic spacer existed.

It is well known that 6FDA produces a soluble polyimide in combination with most aromatic diamines because of the effect of two trifluoro methyl groups. The polyimide based on 6FDA/12G1-AG-Terphenyldiamine was readily soluble in NMP, however 12G2-AG-Terphenyldiamine monomer itself was insoluble in NMP. Therefore, THF that have high affinity for hydrocarbon moiety was added, and the polyimide based on 6FDA /12G2-terphenyldiamine was eventually obtained in NMP/THF (1/1, volume ratio), using the same conditions as two-step polymerization systems in NMP (Table 1).

### 3.3. Polymer characterization and properties

The solubility in various organic solvents of the obtained soluble polyimides are summarized in Table 2. It is interesting that the solubility of these dendronized polyimides were dramatically changed and these were insoluble or less soluble in polar aprotic solvents such as NMP, DMF and DMSO, but easily soluble in less polar solvents such as dichloromethane, chloroform, toluene and THF. This solubility change is probably affected by hydrocarbon moieties of dendritic side chains of polyimides.

**Figure 5.**
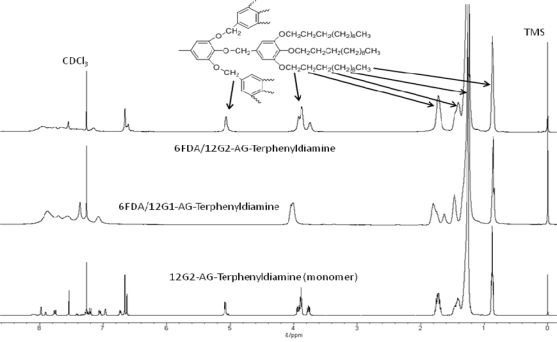
Representative ^1^H NMR spectrum of dendronized polyimides.

As four dendronized polyimides out of the seven soluble dendronized polyimides obtained from these experimental are soluble in chloroform (Table 2), ^1^H-NMR measurements using CDCl_3_ as a solvent and SEC measurements using chloroform as an eluent can be performed. For example, Figure 5 shows the ^1^H-NMR spectra of the polyimides based on 6FDA/12G2-AG-Terphenyldiamine, 6FDA/12G1-AG-Terphenyldiamine, and the monomer, 12G2-AG-Terphenyldiamine. As ^1^H-NMR spectra of polyimides shows broad peaks characteristic of a polymer NMR spectrum, the polymer formation is consequently confirmed. In the ^1^H-NMR spectrum of the polyimide based on 6FDA/12G2-AG-Terphenyldiamine, broad methylene proton peak of Ar-O-C*H*_2_-Ar existing in the second generation of dendoron appears, and the intensity of aromatic protons becomes very low in comparison with the peaks based on nine long-chain alkyl chains. Thus, the structures of dendronized polyimides were confirmed by ^1^H-NMR measurements.

**Figure 6.**
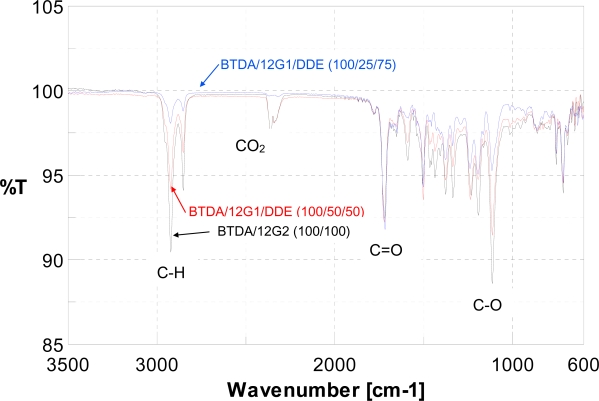
Representative ATR spectrum of dendronized polyimides.

Representative ATR spectrum of dendronized polyimides based on 12G1-AG-Terphenyldiamine and 12G2-AG-Terphenyldiamine were shown in Figure 6 and these spectrum show the strong absorptions based on C-H bonds of long-chain alky groups and the strong absorptions of C-O bonds of alkyloxy groups, and these absorption intensities become stronger with the increase of long-chain alkyl ether segments in the polyimides.

**Figure 7.**
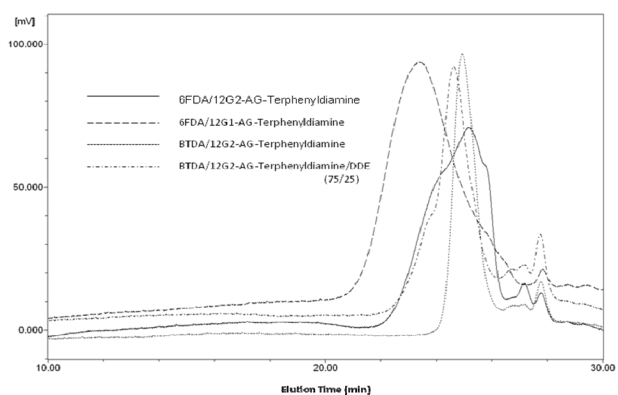
Representative SEC traces of dendronized polyimides.

The results of SEC measurements in chloroform as an eluent are summarized in Table 1 and SEC traces are shown in Figure 7. The inherent viscosities of four dendronized polyimides, soluble in chloroform, were not high, in the range of 0.06–0.28 dLg^−1^. Therefore, the weight average molecular weights determined SEC measurements were also not so high, in the range of 12,800–43,600. Although the molecular weights were not so high, SEC traces indicates the formation of polymers, because these SEC traces display typical molecular weight distributions (Mw/Mn = 1.3–1.9) for polycondensation polymers. In addition, peaks around 27–28 min (elution time) considered as un-reacted monomers are very small.

**Figure 8.**
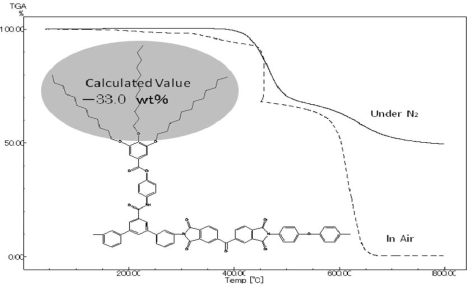
Representative TGA traces of dendronized polyimides based on 12G1-AG-Terphenyldiamine {(BTDA/12G1-AG-Terphenyldiamine/DDE (100/50/50)}.

**Figure 9.**
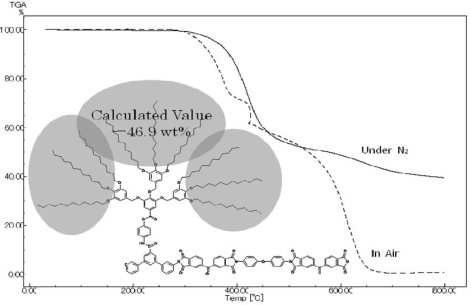
Representative TGA traces of dendronized polyimides based on 12G2-AG-Terphenyldiamine {(BTDA/12G2-AG-Terphenyldiamine/DDE (100/50/50)}.

The glass transition temperatures (Tg) of these dendronized polyimides were determined by DSC measurements, and the thermal stabilities were evaluated in terms of 10% weight-loss temperatures (Td_10_) in TGA measurements. The data are summarized in Table 3, and the representative TGA traces are shown in Figure 8 and Figure 9. It is sometimes difficult to recognize Tg of rigid polymers such as polyimides. Therefore, the DSC measurements were performed at the higher heating rate (20 °C/min) and using relatively much film samples (30 mg) to read heat capacity jump easier, referring the previous papers [41,50].

As can be seen from Table 3, the Tg values of these polyimides that were recognized above measurements were in the range from 249–311 °C, and are 100–150 °C lower than those of the conventional fully aromatic polyimides, however, are 100–150 °C higher than the commodity thermoplastics. Consequently, these soluble dendritic polyimides based on 12G1-AG-Terphenyldiamne and 12G2-AG-Terphenyldiamine can be ranked as heat resistant polymers.

The Td_10_ values of these dendronized polyimides bearing long-chain alkyl groups in Table 3 were in the range 349~455 °C in air and 375~449 °C under nitrogen, showing similar values observed in soluble polyimides obtained from our laboratory (*ca.* 400~500 °C). Although these Td_10_ values of the polyimides obtained in our laboratory are 100~200 °C lower than those of wholly aromatic polyimides; however, these soluble dendritic polyimides based on 12G1-AG-Terphenyldiamne and 12G2-AG-Terphenyldiamine still can be ranked as heat resistant polymers. Figures 8 and 9 show the TGA traces of dendronized polyimides based on BTDA/12G1-AG-Terphenyldiamine (100/50/50), BTDA/12G2-AG-Terphenyldiamine (100/50/50), respectively. These TGA traces showed steep weight loss at the intial stage of degradation, and these weight loss percent almost correspond the calculated value of the weight percent of alkyl groups in polymer segments. Therefore, it is considered that the degradation of long-chain alkyl groups occurred at the initial stage of thermal degradation. Furthermore, these TGA traces also show the evidence that the long-chain alkyl groups exist in the polyimides and the cleavage of alkyl groups did not occurred during the polymerization.

### 3.4. Alignment layer application for VAN-LCDs

**Figure 10.**
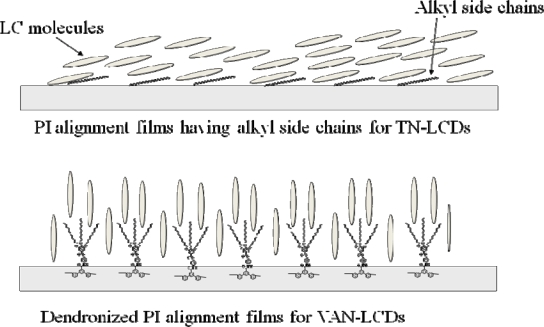
Vertical alignment of LC molecules using dendronized polyimides as alignment layers.

The alignment layer application for VAN-LCDs using polyimides having dendritic side chains was performed at Cheil Ind. Inc., Korea. The polyimide alignment layers containing 8 mol % of 12G1-AG-Terphenyldiamine were utilized for the vertical alignment mode (VA-mode). The synthesis of polyimide alignment layers containing 8 mol % of 12G1-AG-Terphenyldiamine was carried out in NMP as a solvent by conventional two step polymerization method regularly used for the synthesis of polyimide alignment layers for TN-LCDs, and 12G1-AG-Terphenyldiamine monomer was used as one of the diamine components. LCDs test cell properties are summarized in Table 4. PIA-DEN represents the test cell using the polyimide alighnment layers containing 8 mol % of 12G1-AG-Terphenyldiamine, and TN represents the test cell using the regular polyimide alignment layers. The pretilt angles of LC molecules were over 89° in PIA-DEN test cells, which are the suitable values for VAN-LCDs. It is speculated that an extremely bulky and hydrophobic dendritic moieties affects the generation of pretilt angles between the surface of polyimide and liquid crystalline molecules as illustrated in Figure 10. The considerably lower surface energy value of the PIA-DEN alignment film in comparison with the one of TN mode also indicate that the surface of polyimides containing dendritic moieties is more hydrophobic.

**Figure 11.**
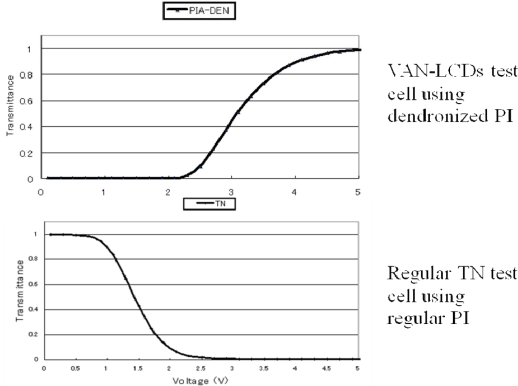
Voltage-transmittance curves of LCD test cells using dendronized and conventional polyimides.

The various important properties of PIA-DEN test cells such as voltage holding ratio (VHR), response time, contrast ratio, residual DC, and image sticking are equivalent or advantageous in comparison with those of regular TN test cell. Figure 11 shows a V-T (voltage-transmittance) curve of these test cells, and shows a dramatic change of T. Consequently, it is convinced that the dendritic monomers, and dendritic polyimides developed by our research can be applied for the alignment films for VAN-LCDs.

## 4. Conclusions

The novel dendritic diamines monomers bearing long-chain alkyl groups (12G1-AG-Terphenyldiamine, 12G2-AG-Terphenyldiamine) were successfully synthesized. The characteristic future of synthetic methods is that Suzuki coupling reaction is applied for the connection of aminophenyl groups. This method is considered the versatile synthetic method for aromatic diamine monomer synthesis because the reduction of corresponding nitoro compound, sometime cause a side-reaction, is not nessesary. The corresponding soluble dendritic polyimides were obtained by polycondensation with tetracarboxylic dianhydride monomers such as benzophenone tertracarboxylic dianhydride (BTDA). The common two-step polymerizations in NMP are difficult; however, the expected dendritic polyimides can be obtained in aromatic polar solvents such as *m-*cresol and pyridine. It is speculated that the hydrocarbon and phenyl moiety of dendritic diamine monomers reduces the solubility of polyimides in NMP, but raise the solubility in aromatic polar solvents. The solubility of these dendoronized polyimides is characteristic; soluble in common organic solvents such as dichloromethane, chloroform, toluene and THF. The hydrocarbon moieties of dendritic segments may help the increment of solubility in these solvents. These dendronized polyimides exhibited high glass transition temperatures and good thermal stability in both air and under nitrogen. According to TGA measurements, it is considered that the thermal decompositions of long-chain alkyl groups firstly occur at the initial stage of decomposition. Their application as alignment layers for LCDs was investigated, and it was found that these polyimides having dendritic side chains were applicable for the vertically aligned nematic liquid crystal displays (VAN-LCDs). It is speculated that an extremely bulky and hydrophobic dendron moiety affects the generation of vertical alignment.

## Figures and Tables

**Table 1.** Polyimides and copolyimides based on 12G1-AG-Terphenyldiamine, 12G2-AG-Terphenyldiamine.

**Monomer**			**Polymerization solvent**	**Poly(amic acid)**	**Polyimide**				
**Dianhydride^a^, ^b^**	**Diamine^c^**	**Comonomer^d^**		**η_inh_^i^**	**Solubility^j^**	**η_inh_^i^**	**Molecular Weight^k^**		
		
	**(mol%)**	**(mol%)**		**dLg^−1^**		**dLg^−1^**	**Mn**	**Mw**	**Mw/Mn**
BTDA	12G1		NMP^e^	0.31	Insoluble				
BTDA	12G1 (50)	DDE (50)	NMP	1.00	Insoluble				
BTDA	12G1		*m*-cresol^f^		Insoluble				
BTDA	12G1 (75)	DDE (25)	*m*-cresol		Insoluble				
BTDA	12G1 (50)	DDE (50)	*m*-cresol		Soluble	0.56			
BTDA	12G1 (25)	DDE (75)	*m*-cresol		Soluble	0.78			
BTDA		DDE	*m*-cresol		Insoluble				
BTDA	12G2		Pyridine^g^		Soluble	0.06	11700	12800	1.3
BTDA	12G2(75)	DDE (25)	Pyridine		Soluble	0.12	16500	20800	1.3
BTDA	12G2 (50)	DDE (50)	Pyridine		Soluble	0.22			
BTDA	12G2 (25)	DDE (75)	Pyridine		Insoluble				
6FDA	12G1		NMP	0.36	Soluble	0.28	22200	43600	1.9
6FDA	12G2		NMP/THF^h^		Soluble	0.12	12400	20100	1.6

a BTDA: 3.3′,4,4′-benzophenonetetracarboxylic dianhydride.

b 6FDA: 4,4′-hexafluoroiso-propylidenediphthalic anhydride.

c See Figure 3.

d DDE: 4, 4′-diaminodiphenyl ether.

e Reaction condition: r.t., 12 h for poly(amic acid)s, pyridine (5 moles)/Ac_2_O (4 moles), 120 °C, 4 h for polyimides.

f Reaction condition: 80 °C, 2 h → 160 °C, 8 h.

g Reaction condition: 40 °C, 12 h → Ac_2_O (4 moles), 120 °C, 4 h.

h Reaction condition: r.t., 12 h → pyridine (5 moles)/Ac_2_O (4 moles), 120 °C, 4 h.

i Measured at 0.5 g dL^−1^in polymerization solvent at 30 °C.

j Solubility in polymerization solvent during polymerization (imidization).

k Determined by SEC in CHCl_3_ calibrated with a series of polystyrenes as a standard.

**Table 2.** Solubility of polyimides and copolyimides based on 12G1-AG-Terphenyldiamine, 12G2-AG-Terphenyldiamine.

**Polymer Compotition**			**Solubility**								
**Dianhydrides**	**Diamine**	**Comonomer**	**Solubility in various solvents (5 wt%)^a^**								
	
	**(mol %)**	**(mol %)**	**NMP**	**DMF**	**DMSO**	**CH_2_Cl_2_**	**CHCl_3_**	**THF**	**Toluene**	**Pyridine**	**m-Cresol**
BTDA	12G1 (50)	DDE (50)	S(h)	I	I	I	I	I	I	I	S(h)
BTDA	12G1 (25)	DDE (75)	S(h)	I	I	I	I	I	I	I	S(h)
BTDA	12G2		I	I	I	S(h)	S	S	S	S(h)	S(h)
BTDA	12G2(75)	DDE (25)	PS(h)	I	I	S	S	S	S	S(h)	S(h)
BTDA	12G2 (50)	DDE (50)	PS(h)	I	I	I	I	I	I	S(h)	S(h)
6FDA	12G1		S	S	I	PS	S	S	S	S	S
6FDA	12G2		I	I	I	S	S	S	S	S	S

a S, soluble; S(h), soluble after heating; PS, partially soluble; PS(h), partially soluble after heating; I, insoluble.

**Table 3.** Thermal properties of polyimides and copolyimides based on 12G1-AG-Terphenyldiamine, 12G2-AG-Terphenyldiamine.

**Polymer Compotition**			**Thermal Property**		
**Dianhydrides**	**Diamine**	**Comonomer**	**Tg^a^**	**Td_10_^b^**	
		
	**(mol%)**	**(mol%)**	°**C**	°**C in Air**	°**C in N_2_**
BTDA	12G1 (50)	DDE (50)	not observed	455	449
BTDA	12G1 (25)	DDE (75)	not observed	442	448
BTDA	12G2		249	365	381
BTDA	12G2(75)	DDE (25)	not observed	362	384
BTDA	12G2 (50)	DDE (50)	269	349	386
6FDA	12G1		311	436	440
6FDA	12G2		271	441	375

a Measured by DSC at a heating rate of 20 °C/min in N_2_ on second heating.

b 10% weight loss temperature measured by TGA at a heating rate of 10 °C/min.

**Table 4.** LCDs test cell properties using the alignment films containing dendronized polyimides.

**ITEM**		**PIA-DEN**	**TN mode**
Pretilt angle (°)		>89	4~6
Surface energy (dyn/cm^2^)^a^		39	48
VHR (%)	25°C	>99	>99
	60°C	>98	>95
Response time (ms)		<25	<30
Contrast ratio		580	250
Residual DC (mv)		<200	<200
Image sticking		<1	<1

a Surface energy of polyimide alignment films measured by a contact angle metod.
